# Beneficial Effects of Hydrogen-Rich Saline on Early Burn-Wound Progression in Rats

**DOI:** 10.1371/journal.pone.0124897

**Published:** 2015-04-13

**Authors:** Song Xue Guo, Yun Yun Jin, Quan Fang, Chuan Gang You, Xin Gang Wang, Xin Lei Hu, Chun-Mao Han

**Affiliations:** 1 Department of Burns, Second Affiliated Hospital, School of Medicine, Zhejiang University, Hangzhou, Zhejiang, China; 2 Department of Plastic Surgery, Binjiang Branch, Second Affiliated Hospital, School of Medicine, Zhejiang University, Hangzhou, Zhejiang, China; 3 Department of Orthopaedics, Binjiang Branch, Second Affiliated Hospital, School of Medicine, Zhejiang University, Hangzhou, Zhejiang, China; The University of Tokyo, JAPAN

## Abstract

**Introduction:**

Deep burn wounds undergo a dynamic process known as wound progression that results in a deepening and extension of the initial burn area. The zone of stasis is more likely to develop more severe during wound progression in the presence of hypoperfusion. Hydrogen has been reported to alleviate injury triggered by ischaemia/reperfusion and burns in various organs by selectively quenching oxygen free radicals. The aim of this study was to investigate the possible protective effects of hydrogen against early burn-wound progression.

**Methods:**

Deep-burn models were established through contact with a boiled, rectangular, brass comb for 20 s. Fifty-six Sprague-Dawley rats were randomly divided into sham, burn plus saline, and burn plus hydrogen-rich saline (HS) groups with sacrifice and analysis at various time windows (6 h, 24 h, 48 h) post burn. Indexes of oxidative stress, apoptosis and autophagy were measured in each group. The zone of stasis was evaluated using immunofluorescence staining, ELISA, and Western blot to explore the underlying effects and mechanisms post burn.

**Results:**

The burn-induced increase in malondialdehyde was markedly reduced with HS, while the activities of endogenous antioxidant enzymes were significantly increased. Moreover, HS treatment attenuated increases in apoptosis and autophagy postburn in wounds, according to the TUNEL staining results and the expression analysis of Bax, Bcl-2, caspase-3, Beclin-1 and Atg-5 proteins. Additionally, HS lowered the level of myeloperoxidase and expression of TNF-α, IL-1β, and IL-6 in the zone of stasis while augmenting IL-10. The elevated levels of Akt phosphorylation and NF-κB p65 expression post burn were also downregulated by HS management.

**Conclusion:**

Hydrogen can attenuate early wound progression following deep burn injury. The beneficial effect of hydrogen was mediated by attenuating oxidative stress, which inhibited apoptosis and inflammation, and the Akt/NF-κB signalling pathway may be involved in regulating the release of inflammatory cytokines.

## Introduction

Burn wounds are not static and present an evolutionary progression in which the existing wound tissue deepens and expands via multiple pathophysiological mechanisms during the first few days after the burn injury[1, 2]. During this progression, superficial partial-thickness burns can convert to deep partial-thickness or full-thickness, and initially unburned skin tissue surrounding the burn wound can also be included in the burn scale with progressive damage. Jackson DM[3] was the first to suggest the theory of three concentric zones of burn wound tissue, comprising the coagulation, stasis and hyperaemia zones. Unlike the coagulation zone, which is characterised by direct thermo-induced irreversible necrosis, the stasis zone is considered potentially salvageable, although it carries the risk of hypoperfusion, which can cause progressive necrosis during the early stages of burn injuries[2]. The hyperaemia zone carries less risk of necrosis and usually recovers well. Overall, wound progression may result in hypertrophic scarring, wound contractures, infections, sepsis and mortality without appropriate intervention[4]. Therefore, preventing wound progression is a challenge for clinical therapy.

The mechanisms of burn wound progression include vasoconstriction/vasodilation, injury by oxygen free radicals, hypoperfusion and microthrombosis, all of which result in inflammatory cascade activation (signalling the activation and release of inflammatory cytokines) and even cell death (necrosis or apoptosis)[1, 2, 5]. Autophagy, previously regarded as type II programmed cell death, has also been reported to be involved in early wound progression after a burn, although it is unclear whether this response is beneficial or detrimental [6, 7].

The small gas molecule hydrogen (H_2_) is able to penetrate the cell membrane and enter the cytosol, mitochondria, and nucleus and selectively scavenge hydroxyl radicals (·OH) and peroxynitrite anions (ONOO^-^) of reactive oxygen species (ROS), thereby preventing their interference in normal metabolism and signal transmission[8]. Moreover, H_2_ has protective effects on several types of oxidative stress-induced organ damage, such as early brain injury after subarachnoid haemorrhage (SAH), radiation-induced injury, sepsis, and ischaemia-reperfusion injury in multiple organs, by suppressing oxidative stress and apoptosis, inhibiting inflammatory cell infiltration and regulating pro-inflammatory cytokine expression and inflammation/apoptosis-related signalling pathways[9–14]. With respect to skin wounds, previous in vitro and in vivo studies have indicated that H_2_ could protect skin flap or diabetic wounds from ischaemia/reperfusion or hyperglycaemia-induced injuries, respectively[15, 16]. Hence, we hypothesised on the possible impacts of H_2_ on burn wound progression, and all experiments were designed to evaluate potential mechanisms of action and regulation. Following our prior experiences and previous work, we selected hydrogen-rich saline (HS) as a carrier of molecular hydrogen via intraperitoneal (IP) administration as an effective and convenient therapy in this study.

## Materials and Methods

### Animal model and grouping

This study was performed according to protocols approved by the Zhejiang University Committee on Animal Care and Use and strictly followed the National Institutes of Health Guidelines for the Care and Use of Laboratory Animals. Adult male Sprague-Dawley rats (weighing approximately 250–300 g) were purchased from the Animal Centre of Zhejiang Traditional Medical University (Hangzhou, China) and housed with a 12-hour light/dark cycle in a filtered-air unit at a constant temperature and humidity and free access to food and water. All animals were randomly assigned to seven groups, including the sham group (n = 8), three burn+vehicle (saline, 10 ml/kg intraperitoneally) groups and three burn+hydrogen saline (10 ml/kg intraperitoneally administered immediately and every 12 h post-operation) groups and sacrificed at 6 h, 24 h, and 48 h post burn (n = 8 in each group). The burn model was induced in accordance with previous reports [6, 17–19]. A custom-made rectangular brass comb (with a transverse section of approximately 18 mm×10 mm) was boiled in 100 ºC water for 5 min and then applied to the shaved skin surface of the rat dorsum for 20 s after anaesthesia had been induced (sodium pentobarbital, 50 mg/kg intraperitoneally). The breath and heart rates of the experimental rats were monitored during anaesthesia. A row of four bands of full-thickness burns (20 mm×10 mm) was created with three interspaces of uninjured skin (20 mm×5 mm), with the interspaces representing the zone of stasis according to prior studies[6, 18, 19]. The burn wound area represented approximately 4% of the total body surface area. For the sham group, the brass comb was heated in 25 ºC water and applied without saline or hydrogen saline application after anaesthesia. The animals in all groups were sacrificed by intraperitoneal administration of a pentobarbital overdose at various time points. One band of the interspace skin with 2 mm of burned tissue on each side (20 mm×9 mm) was harvested from each burn model and stored in 10% formalin at 4 ºC for subsequent immunohistochemistry analysis, while the remaining two bands of interspace skin (without burn tissue; 20 mm×5 mm) from each burned rat were stored at -80 ºC for ELISA, and Western blot assays. Three corresponding areas of unburned skin from each rat of the sham group were used as controls.

### HS preparation

HS was prepared as described previously[14, 20–22]. Hydrogen gas was dissolved in 0.9% saline for 6 h under 0.4 MPa pressure to a create a supersaturated solution using a HS-producing apparatus from the Department of Diving Medicine, the Second Military Medical University, Shanghai, China and Hydrovita Beverage Co., Ltd., Beijing, China. Gas chromatography (Biogas Analyser Systems-1000, Mitleben, Osaka, Japan) was applied to monitor the concentration of hydrogen (maintaining it above 0.6 mmol/L) in the HS following the methods of previous articles[8]. Prepared HS was stored under atmospheric pressure at 4–8 ºC in an aluminium bag with no dead volume and was sterilised by gamma radiation.

### Histological preparation

Fixed skin samples were embedded in paraffin and then sectioned at a thickness of 5 μm using a Rotary Microtome (RM2245, Leica, Solms, Germany). Prior to staining, all slices were deparaffinised and rehydrated.

### Hematoxylin and eosin staining

Hematoxylin and eosin (HE) staining was performed; the tissue slices were observed under the microscope (DM2500, Leica, Solms, Germany); and images were recorded. Five high-magnification files were selected randomly for observation from every slice.

### TUNEL staining for apoptosis

The staining operation was performed with a commercial cell death detection kit purchased from Roche Diagnostics (Indianapolis, USA) according to the manufacturer’s protocol. The stained slices were observed by microscopy (DM2500, Leica, Solms, Germany), and images were recorded. The index of apoptosis was calculated as the percentage of apoptotic cells among all cells counted under blinded conditions. Cells with dark brown-stained nuclei were counted by two independent investigators who were blinded to the group assignments. At least three visual fields per slide and five slides per group were evaluated by the investigators.

### Immunohistochemical staining

The slices from paraffin-embedded tissues were subjected to immunohistochemical staining for myeloperoxidase (MPO). The prepared slices were washed in PBS for 10 min and then boiled in 0.01 mmol citrate buffer (pH = 6) for 10 min for antigen retrieval. After incubation with hydrogen peroxide for 10 min, 5% bovine serum albumin (BSA) was applied as the blocking solution for 20 min at room temperature. Without washing, the sections were incubated with anti-myeloperoxidase antibody (1:100)(ab9535, Abcam, Cambridge, UK) overnight at 4 ºC After being rinsed with PBS, the sections were incubated with goat anti-rabbit secondary antibody (1:500)(BA1054, Boster, Wuhan, China) and then visualised using a 3,3-diaminobenzidine (DAB) kit (AR1022, Boster, Wuhan, China). Finally, the sections were examined, and images were recorded using a microscope at 400× magnification (DM2500, Leica, Solms, Germany).

### Oxidative stress assessment

Lipid peroxidation was evaluated by detecting malondialdehyde (MDA) levels at various time points (6 h, 24 h, 48 h) post burn. Skin tissue homogenates from the burn wounds reacted with a thiobarbituric acid reactive species (TBARS) assay kit (KGT003-1, KeyGEN Biotech, Nanjing, China) to determine the MDA levels, which were expressed in nmol/mg protein. Tissue superoxide dismutase (SOD), glutathione peroxidase (GSH-Px) and catalase (CAT) activities were evaluated to determine the oxidative stress status in the skin tissues of burn wounds and were measured using commercial assay kits from KeyGEN Biotech (SOD with KGT00150, GSH-Px with KGT-014, CAT with KGT017, Nanjing, China) according to the manufacturer's protocols. The results were expressed in U/mg protein for SOD and CAT and in nmol/min/mg protein for GSH-Px. Absorbance values were measured using a microplate reader (Model 680 Microplate Reader, BIO-RAD, CA, USA). The frozen tissue samples from biopsies in rats were crushed, ground and then homogenised using a tissue homogeniser with homogenisation buffer (4 ºC), which included 1 mmol/L phenylmethylsulfonyl fluoride (PMSF), 1 mg/ml pepstatin A, 1 mg/ml aprotinin, and 1 mg/ml leupeptin in phosphate-buffered saline solution (pH 7.2) containing 0.5% sodium deoxycholate and 1% Triton X-100, at a ratio of 100 mg tissue/ml. After incubation at 4 ºC for one hour, the final homogenate was centrifuged at 10 000 g for 15 min at 4 ºC, and the supernatants were used for detection kits. The total protein concentration was determined using the BCA assay kit (KGPBCA, KeyGEN Biotech, Nanjing, China).

### Enzyme linked immunosorbent assay (ELISA)

The skin tissue levels of MPO were quantified by rat-specific commercial ELISA platinum kits purchased from Lianshuo Biological Technology (DRE60365Ra, Shanghai, China), according to the manufacturer’s protocols. The skin homogenate was obtained via the method abovementioned. The results were expressed in ng/mg protein.

### Western blot analysis

Skin samples were collected from the rats for Western blot analysis. Briefly, frozen skin samples were cut into pieces and lysed with RIPA lysis buffer (AR0105, Boster, Wuhan, China) for one hour on ice, and the lysates were centrifuged at 14000 g for 10 min. After being mixed with loading buffer, the protein samples were subjected to SDS-PAGE and transferred onto nitrocellulose membranes by electrophoresis, while aliquots of samples were used to determine the protein concentration of each sample using a bicinchoninic acid (BCA) kit (KGPBCA, KeyGEN Biotech, Nanjing, China). Subsequently, membranes were incubated in blocking buffer for two hours and incubated overnight at 4°C with the following primary antibodies: anti-TNF-α antibody (SC-1351, Santa Cruz Biotechnology, CA, USA), anti-IL-1β antibody (SC-23460, Santa Cruz Biotechnology, CA, USA), anti-IL-6 antibody (SC-1265, Santa Cruz Biotechnology, CA, USA), anti-IL-10 antibody (ab9969, Abcam, Cambridge, UK), anti-cleaved caspase-3 antibody (1:500)(SC-22171-R, Santa Cruz Biotechnology, CA, USA), anti-Bcl-2 antibody (1:800)(SC-783, Santa Cruz Biotechnology, CA, USA), anti-Bax antibody (1:500)(SC-493, Santa Cruz Biotechnology, CA, USA), anti-NF-κB p65 antibody (1:500)(ab16502, Abcam, Cambridge, UK), anti-p-Akt antibody (1:300)(SC-33437, Santa Cruz Biotechnology, CA, USA). β-actin (1:2000)(SC-47778, Santa Cruz Biotechnology, Santa Cruz, CA) was used as a control on the same membranes. After applying secondary antibodies, the bands were detected with West Dura Extended Duration Substrate (Pierce, USA) and x-ray film (Kodak, USA) and then analysed by Bandscan 5.0 software via comparison with β-actin.

### Statistical analysis

Data were presented as the mean±standard error of the mean (SEM). GraphPad Prism version 5.01 (San Diego, CA, USA) was used for statistical analysis. The comparisons between sham group and each of burn groups were conducted with Mann-Whitney U test. Multiple comparisons among burn groups which were treated with saline or HS at different time points were analysed with two-way analysis of variance (ANOVA) followed by Bonferroni posthoc test. A value of P<0.05 was accepted as statistically significant.

## Results

### Histological findings

**Fig 1:**
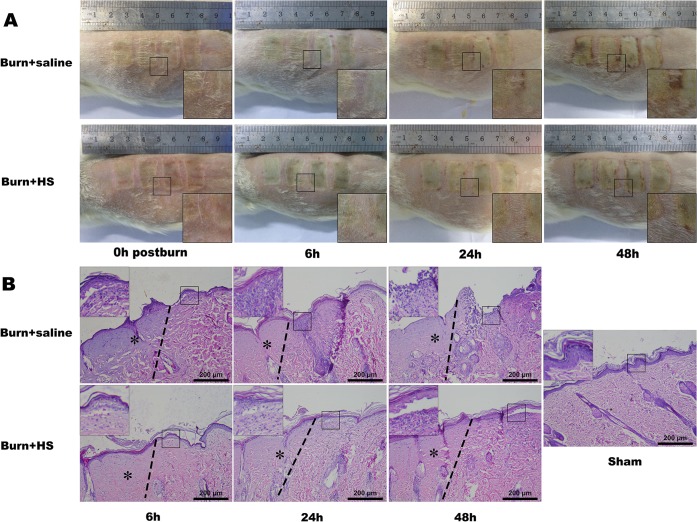
Representative clinical images and HE staining of burn models with saline and HS treatment. The interspaces between two burn zones became narrower and were inclined to merge in the burn+saline groups over time, whereas the interspaces remained relatively stable at various time points with HS treatment (small and amplified square frames) (A). The dotted line indicated the progression boundary, and the regions labelled by asterisks presented the obvious hyalinization, which received direct thermal injury. Some changes in the zone of stasis (right part aside dotted line), such as disturbed constructions of the epidermis and inflammatory cell infiltration (small and amplified square frames), can be observed in the burn+saline groups, whereas HS treatment improved these changes at the late time points (24 h and 48 h) (B) (100× magnification). The sample size was n = 8 for each group.

Gross sections of various burn+saline groups revealed that the interspaces between two burn wounds gradually narrowed and presented a tendency to merge following the burn, whereas the interspaces remained relatively stable at various time points in the HS groups (Fig 1A). Certain characteristics, such as severe epidermis layer thinning, epithelium nuclei elongation, and dermis layer swelling with collagen alteration, could be observed in the burn+saline groups, whereas HS treatment alleviated these changes over time (Fig 1B).

### HS ameliorates oxidative stress in the stasis zone of rat burn wounds

**Fig 2:**
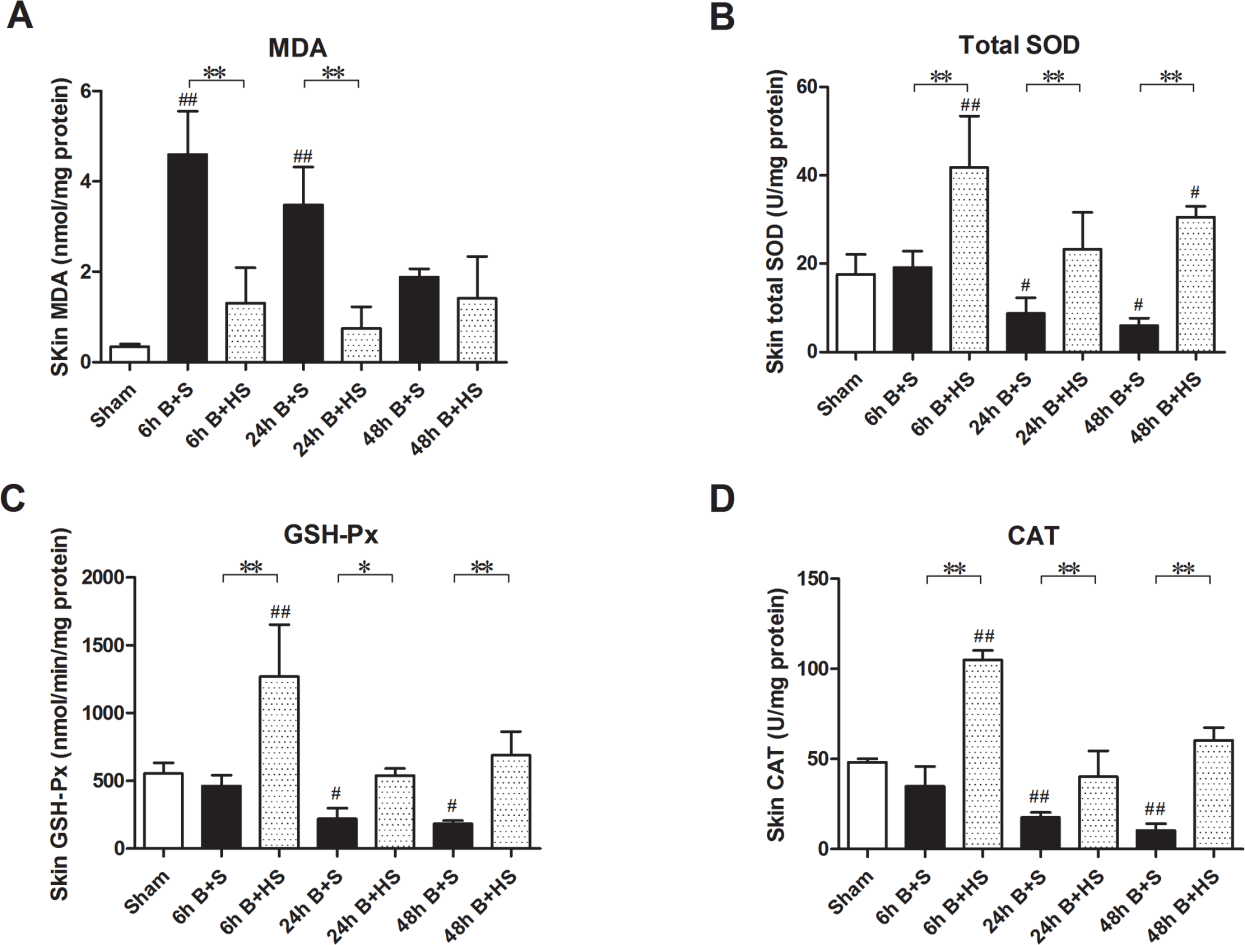
Assessment of oxidative stress within the zone of stasis and the effects of HS. The levels of MDA in the interspaces of various burn+saline groups presented remarkable augmentations after burn, which coincided with obvious declines in the activities of endogenous antioxidant enzymes (SOD, GSH-Px, CAT). Application of HS postburn led to the observation that significant reductions in the MDA levels and increases in the activities of antioxidant enzymes appeared at a range of time windows. The sample size was n = 6 for each group. The results were expressed as the mean±SEM.*p<0.05, **p<0.01, versus burn+saline; #p<0.05, ##p<0.01, versus sham. B+S, burn+saline; B+HS, burn+hydrogen-rich saline.

An imbalance between oxidative and antioxidant effects contributes to oxidative stress in vivo and results in severe oxidative damage via various free radicals, such as reactive oxygen species (ROS) and reactive nitrogen species (RNS), among others[23]. MDA was considered as a cause of lipid peroxidation and oxidative stress. The severe burn induced obvious increases in the MDA level in rat skin tissue at 6 h and 24 h post burn, while a slight elevation in MDA also appeared at 48 h post burn but was not significant (Fig 2A). HS treatment significantly decreased the remarkable elevations in MDA compared with those of the saline treatment groups at 6 h and 24 h (Fig 2A). In addition, burn injury markedly reduced the activities of inner antioxidant enzymes in the interspaces of wounds at 24 h post burn (Fig 2B–2D). HS application significantly increased antioxidant enzyme activities at various time points post burn (Fig 2B–2D). Taken together, these data indicate that hydrogen can significantly attenuate burn-induced oxidative damage in the wound tissue of rats by inhibiting oxidative stress and increasing the activities of endogenous antioxidant enzymes.

### HS regulates the levels of MPO and inflammatory cytokines in wounds after burn injury

**Fig 3:**
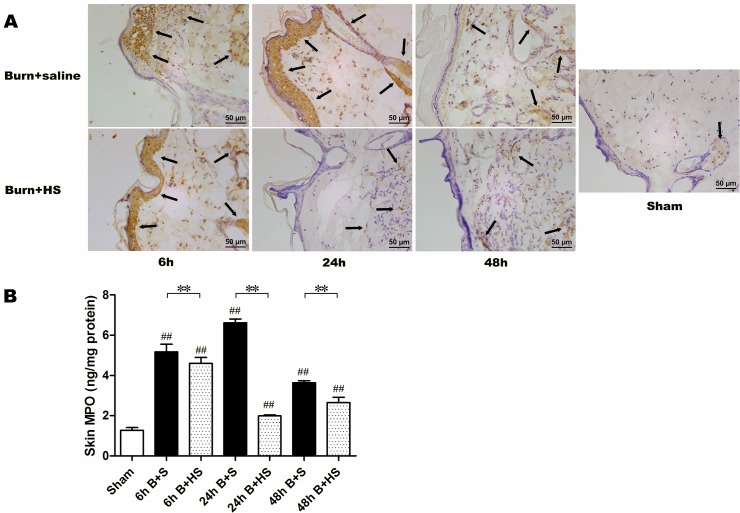
The representative immunohistochemistry staining and ELISA detection of MPO within the zone of stasis. Compared with the sham group, apparent positive staining (brown stained tissues indicated by black arrows) was observed after burn insults at 6 h, 24 h, 48 h (upper row), while HS treatment seemed to decrease the number of positive cells (lower row)(400× magnification). The ELISA detection of MPO in the interspace skin tissues expressed similarly obvious elevations in the MPO level in various burn+saline groups, and a marked effect of HS treatment on decreasing these elevations were also found in the time-paired burn+HS groups (B). The sample size was n = 6 for each group. The results were expressed as the mean±SEM. **p<0.01, versus burn+saline; ##p<0.01, versus sham. B+S, burn+saline; B+HS, burn+hydrogen-rich saline.

**Fig 4:**
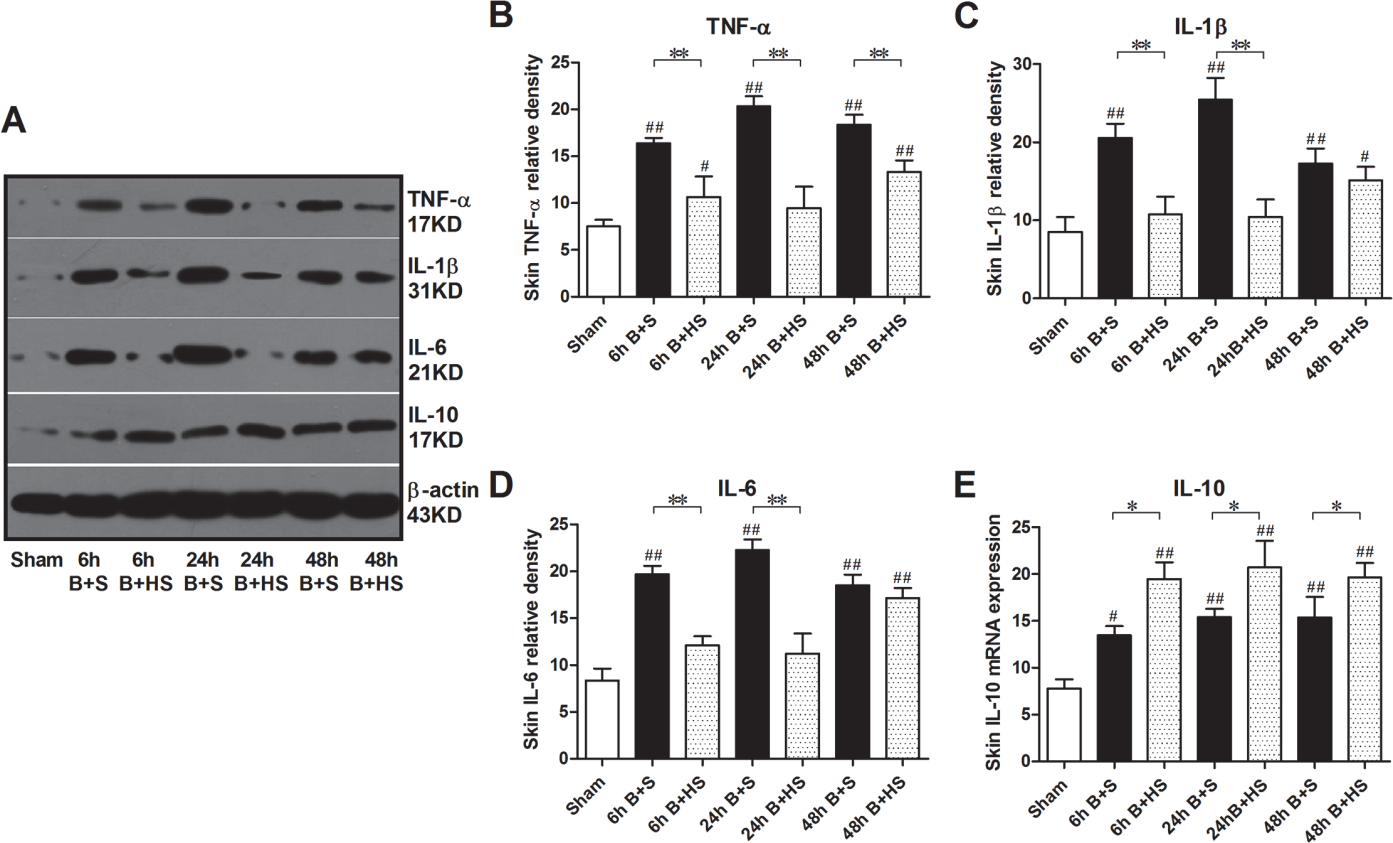
Western blot analysis of inflammatory cytokines in wound interspaces after burn with HS treatment. Parallel increases in TNF-α, IL-1β, and IL-6 protein expression (as proinflammatory cytokines) were found at the three time points via western blot, while IL-10 also increased after burn to mediate the anti-inflammatory response. HS administration reduced the elevations of the three selected pro-inflammatory cytokines, and it further raised the levels of IL-10 to enhance the anti-inflammatory effect in wound tissues. The sample size was n = 6 for each group. The results were expressed as the mean±SEM. *p<0.05, **p<0.01, versus burn+saline; #p<0.05, ##p<0.01, versus sham. B+S, burn+saline; B+HS, burn+hydrogen-rich saline.

MPO, a marker of polymorphonuclear neutrophil leukocyte (PMNL) function and activity, was detected by immunohistochemistry staining and ELISA in skin samples from the burned rats (Fig 3A). HS could decrease the number of positively labelled cells in burned skin tissues at various time points compared with those from other treatment groups at corresponding time points (Fig 3A). Moreover, severe burn injury produced an obvious elevation in MPO levels in the wound skin at 6 h post burn, which then peaked at 24 h. A decline in the elevated levels of MPO in the skin was observed at 48 h, although the level was still significantly higher than that of the sham group. Nevertheless, HS application was able to significantly decrease these burn-induced increases in MPO levels (Fig 3B). With respect to inflammatory cytokines, burn-induced elevations in expression in skin tissue were commonly observed using western blot detection. In particular, compared with the levels in skin tissues from the sham group, the relative expression levels of TNF-α, IL-1β and IL-6 markedly increased at 6 h post burn, and the expression of these mediators similarly peaked at 24 h post burn (Fig 4A–4D). The extent of increased expression was reduced at 48 h after burn injury (Fig 4A–4D). In addition, IL-10 manifested a trend of gradually elevated expression in the burned skin of rats, and all of the expression measurements at the three time points were significantly different from those of the sham group (Fig 4A and 4E). After HS treatment, the relative expression levels of TNF-α displayed marked reduction at various time points compared with the corresponding burn controls (Fig 4A and 4B), while IL-1β and IL-6 showed obvious reduction only at 6 h and 24 h post burn (Fig 4A, 4C and 4D). Furthemore, the elevation of anti-inflammatory cytokines (IL-10) became more significant at all three time pionts (6 h, 24 h,72 h) (Fig 4A and 4E). Taken together, these results show that hydrogen can alleviate the early inflammatory response to burn injury in the cutaneous wound tissues of rats.

### HS reduces burn-induced apoptosis in the cutaneous wound tissues of rats

**Fig 5:**
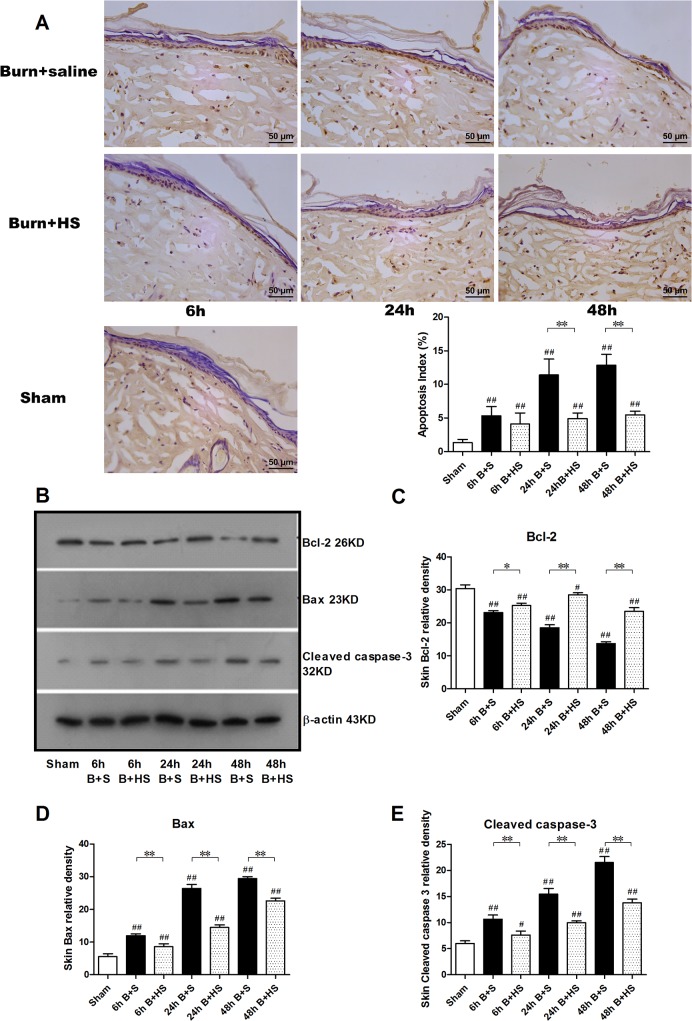
Evaluation of apoptosis in the zone of stasis after burns with HS management. Classic TUNEL staining indicated symbolic brown nuclei that were mainly located in the dermis of the interspace skin postburn (400× magnification)(A). Counting the apoptotic cells indicated a significant elevation in the burn controls over time, with a peak occurring at 72 h post burn. Except for the 6 h group, HS administration effectively decreased the number of apoptotic cells in the treatment groups at 24 h and 48 h (400× magnification)(A). Bcl-2 expression was downregulated after burn insult, while the protein expression of Bax and caspase-3 exhibited increasing trends over time (B-E). Nevertheless, HS seemed to reverse these of expression tendencies at various time points (B-E). The sample size was n = 5 for each group. The results were expressed as the mean±SEM. *p<0.05, **p<0.01, versus burn+saline; #p<0.05, ##p<0.01, versus sham. B+S, burn+saline; B+HS, burn+hydrogen-rich saline.

TUNEL staining was applied to detect apoptotic cells, and positive-labelled cells presented dark brown staining (Fig 5A). The index of apoptosis increased significantly over time after severe burns and was mainly located in the epidermis of the rats (Fig 5A). Western blot analysis confirmed the effect of HS on apoptosis in cutaneous wounds. The protein expression of Bcl-2, which is considered an anti-apoptotic protein, was markedly upregulated with HS treatment at the three time points, although the burn control subjects all presented an obvious decline in protein expression compared with that of the sham groups (Fig 5B and 5C). Correspondingly, Bax, a key component of cellular-induced apoptosis through mitochondrial stress, manifested the opposite trend, i.e., markedly elevated expression of Bax was observed in the various burn controls, whereas HS application significantly downregulated these increases (Fig 5B and 5D). In addition, we investigated the activation of caspase-3, which plays a central role in the execution phase of cell apoptosis. Cleaved caspase-3, the active form of the protein, exhibited a gradual elevation in protein expression over time following a burn, and HS treatment suppressed this increase at the various time points (Fig 5B and 5E). Generally speaking, hydrogen was able to inhibit apoptosis in cutaneous wounds following a burn.

### HS decreases autophagy in rat wounds during the early stages after burn injury

**Fig 6:**
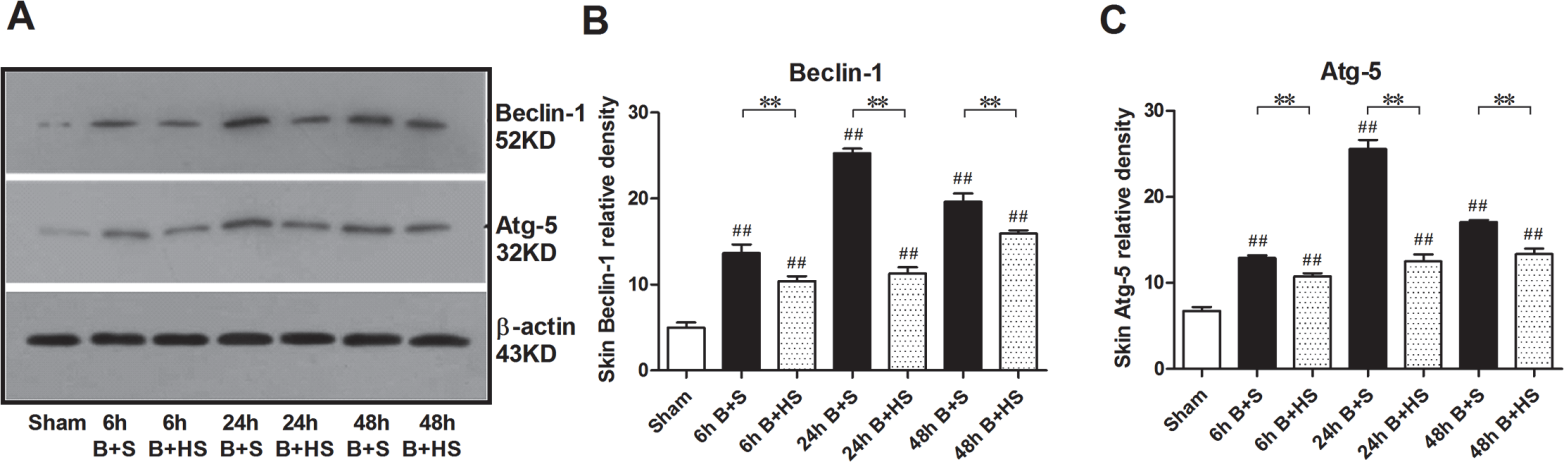
Western blot analysis of autophagy-related proteins in the burn interspace tissues. The expression of Beclin-1 and Atg-5 showed similar increases starting at 6 h post burn and peaking at 24 h, while these remarkable elevations in protein expression decreased slightly at 72 h. HS downregulated these expression increases significantly in various time-paired groups. The sample size was n = 6 for each group. The results were expressed as the mean±SEM. **p<0.01, versus burn+saline; ##p<0.01, versus sham. B+S, burn+saline; B+HS, burn+hydrogen-rich saline.

To evaluate the effect of hydrogen on autophagy in burn wounds, we examined the protein expression of Beclin-1 and Atg-5, which are two key components in the activation of autophagy[24, 25]. The results indicated that the expression levels of Beclin-1 and Atg-5 increased markedly after a burn and peaked at 24 h post burn (Fig 6A–6C). Following HS management, all three groups experienced similar clear declines in the increased expression of Beclin-1 and Atg-5 at the various time points (Fig 6A–6C). Thus, during the early stages post burn, hydrogen was able to inhibit the activation of autophagy in cutaneous wounds.

### HS downregulates NF-κB expression and Akt phosphorylation in the burn interspace tissues of rats

**Fig 7:**
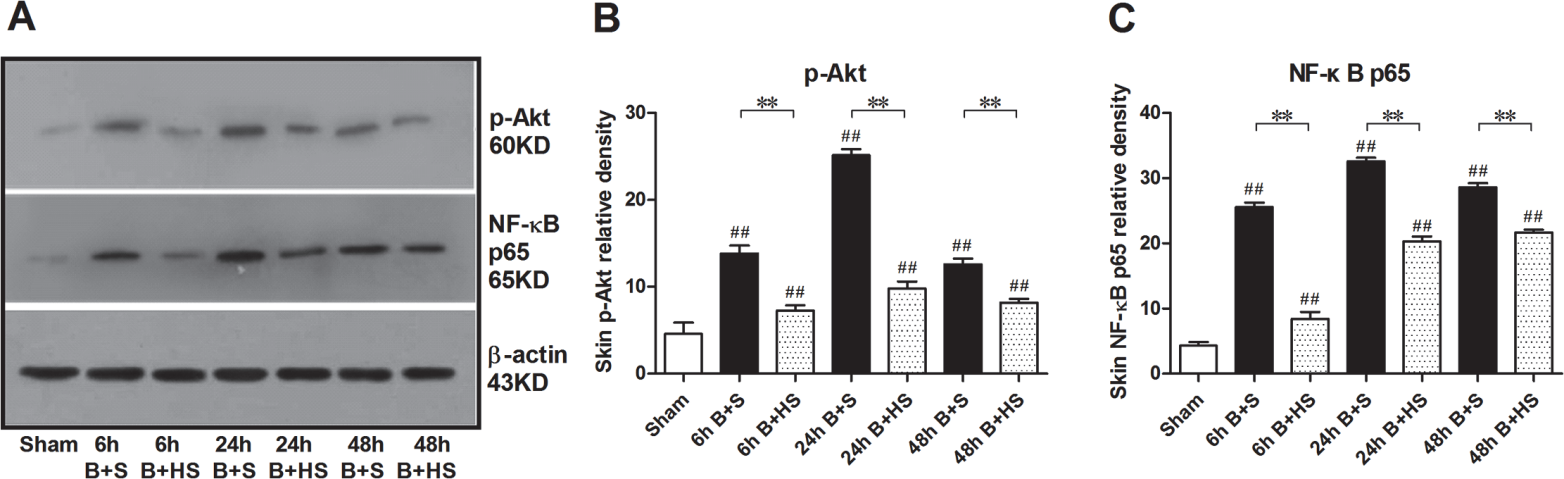
Effect of HS on signals post burn via Western blotting analysis. The phosphorylation of Akt in the zone of stasis increased markedly at the three time windows after burn, and the peak occurred at 24 h. Similarly, the expression of NF-κB p65 underwent a significant elevation post burn. HS application downregulated both the elevation in phosphorylated Akt and that of NF-κB p65 expression. The sample size was n = 6 for each group. The results were expressed as the mean±SEM. **p<0.01, versus burn+saline; ##p<0.01, versus sham. B+S, burn+saline; B+HS, burn+hydrogen-rich saline.

Akt signalling participates in multiple regulation processes associated with apoptosis, autophagy and inflammation[26]. In this study, we observed that the phosphorylation of Akt began to increase at 6 h post burn and peaked at 24 h, at which point, the rate of increase declined compared with that of the sham group (Fig 7A and 7B). Following HS treatment, all groups exhibited a reduction in Akt phosphorylation at all time points (Fig 7A and 7B). Additionally, we investigated the possible regulation of NF-κB signalling in response to HS treatment. Western blot analysis indicated that HS reversed the remarkable upregulation of NF-κB p65 protein expression that occurred in response to burn insults at various time points (Fig 7A and 7C)

## Discussion

Direct thermal insult usually results in irreversible tissue damage within the zone of stasis of burn wounds; however, the extent and area of thermal damage are not restricted to the initial region of injury[1, 2]. Therefore, the periburn zones, including those of stasis and hyperaemia, are at risk of wound progression, which may deepen and extend the existing wound. The skin tissue exhibits early responses to heat stimuli, including direct heat-induced protein denaturation and cell death (necrosis and apoptosis)[1, 2]. Complex factors contribute to further development of the burn wound, such as tissue hypoperfusion, injury through oxygen free radicals and inflammation[1, 2], while autophagy may play a vital role in wound progression via possible mechanisms of survival or nonapoptotic programmed cell death[6, 7]. Moreover, the conversion of burn wounds to full-thickness will result in more severe consequences, such as slow-healing wounds, systemic inflammation, local infection and even sepsis, which lead to a poor prognosis and high risk of mortality[4, 27, 28]. Previous studies have focused on the process of wound progression and effective routines and proper time windows for intervention. Considering its reported effects on oxidative stress, apoptosis and inflammation in multiple disease models, hydrogen may be of value during wound progression[8, 15, 29, 30].

Zhao L et al. stated that hydrogen can protect skin flaps from ischaemia/reperfusion injury by inhibiting inflammation of the local skin tissue[16]. The zone of stasis, which is at risk for further deterioration during wound progression, undergoes similar detrimental effects of hypoxia and ischaemia and is regarded as salvageable with proper and timely intervention[1, 2, 6]. Here, we investigated for the first time the possible preventative effects of hydrogen on transforming the zone of stasis. The results indicate that HS administration significantly attenuates the oxidative stress during early stages in burn wounds by inhibiting membrane lipid peroxidation and enhancing the activity of endogenous antioxidant enzyme systems. In addition, HS treatment markedly inhibited apoptosis and autophagy in the interspace wound tissues after burns. Furthermore, HS application also remarkably decreased protein expression of pro-inflammatory cytokines (TNF-α, IL-1β, IL-6), and upregulated protein expression of the anti-inflammatory cytokine IL-10. The expression of signal proteins, Akt and NF-κB, was significantly repressed after HS management. Taken together, hydrogen may ameliorate burn wound progression by inhibiting oxidative stress, apoptosis and inflammation and regulating the Akt/NF-κB pathway, while the effect on autophagy suppression during wound progression still requires further data.

At the molecular level, the over-production of oxygen free radicals resulting from activation of PMNLs and xanthine oxidase activity mediates oxidative stress-induced tissue and vascular injuries leading to a decrease in endogenous anti-oxidant enzymes (SOD, GSH-Px, CAT, etc.)[1, 2]. As an indicator of oxidative stress, lipid peroxidation may contribute to elevated necrosis in partial-thickness burns[2]. Furthermore, oxidative stress also induces an inflammatory cascade and tissue apoptosis in local wounds and secondary organ injury after burns [1, 2]. As an important component of oxygen free radicals, reactive oxygen species (ROS) are involved in the regulation of autophagy in various cell types and produce distinct effects on cell survival and death[31, 32]. Some researchers have suggested a potential strategy to protect anti-oxidants during post burn management by ameliorating the detrimental impacts of oxidative stress[33–36]. In the present study, H_2_ was able to effectively decrease MDA levels, which is an index of lipid peroxidation, and enhance the activities of endogenous anti-oxidant enzymes. Similar effects have also been validated in a series of previous results involving in vivo and in vitro experiments[33, 35].

As a common physical response to tissue injury, inflammation is activated, including leukocyte margination and infiltration, the release of inflammatory mediators, and the activation of related signals [1]. During the early stages of burn injury, PMNLs are the first leukocytes to arrive at the injury site [1]. Although they play a vital role in removing dead cells and killing bacteria, the overabundance of PMNLs caused by severe burns (which are equal to or exceed those of deep second-degree burns) contributes to exacerbated tissue injury, prolonged wound healing, and even a systemic inflammatory response[1, 2]. Monitoring PMNLs provides a valuable means of evaluating the clinical status of the patient and the effectiveness of interventions. The level of MPO reflects the infiltration of PMNLs, and the burn insult gives rise to an obvious elevation in MPO in burn wounds. Zheng X et al.[30] reported that hydrogen could decrease the ischaemia/reperfusion injury-induced increase in MPO level in the intestine of rats, and a similar effect was also verified in rodent models of brain and lung injuries[29, 37, 38]. Hydrogen also lowered the levels of MPO in the zone of stasis according to our immunohistochemistry and ELISA results. In contrast, inflammatory cytokines attend to the process of inflammation in burn wounds. Secreted by immunocytes, TNF-α, IL-1β and IL-6 are involved in the activation of macrophages, lymphocytes and cytotoxic cells; leukocyte/endothelial adhesion; and the induction of acute-phase protein[1]. In particular, IL-10 is a negative-regulator cytokine generated by immunocytes that antagonises the inflammatory response and regulates autoimmune activity [39]. The tissue level of IL-10 is usually elevated as an intrinsic anti-inflammatory system responding to exogenous stimuli. Gauglitz GG et al. reported obvious increases in serum IL-1, IL-6, IL-10 postburn in rat models[40]. We found that burn insult induced significant augmentation of TNF-α, IL-1β and IL-6 in cutaneous wounds, and IL-10 also displayed a corresponding elevation. Current work from Zhao L et al. indicated that hydrogen is able to reduce TNF-α, IL-1β and IL-6 levels in skin flaps exposed to analogous ischaemia/reperfusion injury within the zone of stasis[16]. In our study, hydrogen downregulated the mRNA expression of TNF-α, IL-1β and IL-6 in the stasis zone while further elevating IL-10. In summary, hydrogen may regulate wound inflammation after burn injuries by influencing PMNLS infiltration and releasing inflammatory cytokines.

Apoptosis, a type of programmed cell death, has been shown to be involved in burn wound progression, exacerbating local ischemic damage and necrosis[41]. Our results and previously reported experimental data indicate similarly marked activation of apoptosis in wound tissue after burns, and it has also been reported that relieving apoptosis can decrease tissue loss and promote burn wound healing[42–45]. Based on its known ability to inhibit oxidative stress-induced apoptosis in lung, heart, and brain tissue, we investigated the effect of hydrogen on wound tissue apoptosis to identify its potential mechanism of action[8, 46, 47]. The results of this study also supported our hypothesis on the ability of hydrogen to inhibit apoptosis. Expression of Bcl-2 protein indicates an anti-apoptotic effect, which can also inhibit autophagy[48]. Combined with the pro-apoptotic protein Bax, the ratio of Bax/Bcl-2 reflects the activation of apoptosis. In addition, burn-induced oxidative stress can prompt phosphorylation of the p38 MAPK signal and further activate caspases, ultimately leading to cell apoptosis[49]. The antioxidant property of hydrogen may influence tissue apoptosis by affecting Bax/Bcl-2 expression and the above-mentioned pathway.

Although we observed an elevated level of autophagy in wounds during the early stage after deep burns, as observed in other reports, and applied HS intervention, we still cannot conclude with certainty that autophagy plays a survival or prodeath role in wound progression postburn. Generally speaking, autophagy is an in vivo pathway that can degrade cellular macromolecule waste via lysosomes and provide the materials necessary to maintain cellular metabolic turnover and homeostasis[50, 51]. This process has also been reported to play dual roles in cell survival and cell death, and the impairment or activation of autophagy is associated with the development of various diseases [52–55]. Autophagy serves a cytoprotective role against various insults, such as infection, ischaemia, and inflammation, among others[56, 57]. However, autophagy may also mediate programmed cell death when it cannot restore tissue cell homeostasis following an insuperable stimulus[58–60]. With regard to burn wound progression, two distinct viewpoints have been offered. Utilising the rat hot-water burn model, Xiao M et al. [13] reported that autophagy plays a role in the prosurvival mechanism against ischaemia and inflammation in the deeper dermis during the later stages (48–72 h) of burn injury, as a complement to apoptosis. Tan J et al.[6] reported the contribution of autophagy towards cell death as similar to that of apoptosis using the comb model, although they were unable to distinguish the beneficial or detrimental effect of autophagy in burn wounds. Although the elevation of autophagy was inhibited markedly by HS, in this study, the levels of autophagy in the zone of stasis at the three time points were still higher than those of normal skin from the sham group. This result did not conform to that of Xiao M et al., and the difference may be attributed to differences in the sample sites. We selected the zone of stasis rather than the directly thermo-injured region (the zone of coagulation) for evaluation. In contrast to the stable necrosis observed in the zone of coagulation, the zone of stasis undergoes wound progression, including cell death, during the early stages post burn (within 48 h). Consequently, we are inclined to assume that autophagy plays a prodeath role during the early stage of burn injuries in conjunction with apoptosis, while hydrogen can decrease this type of cell death. However, these data still do not offer a strongly convincing result to support the beneficial or detrimental effects of autophagy on wound progression, and further studies are needed.

Lastly, the modulation mediated by signalling molecules also attracts our attention to explore potential regulatory targets associated with wound progression. Protein kinase B (Akt) is a crucial node of the PI3K-Akt pathway. The activation of Akt (Akt phosphorylation) can be regarded to act as a cell survival factor by antagonising apoptosis and activating downstream mammalian targets of rapamycin mTOR to inhibit autophagy[61, 62]. In contrast, phosphorylated Akt participates in the activation of NF-κB, which induces the releases of cytokines (such as TNF-α, IL-1β, IL-2, IL-6, IL-8, IL-12, iNOS, and COX2, among others) involved in early immune and inflammatory responses[63–65]. According to our results, the elevation in phosphorylated Akt (p-Akt) postburn was accompanied by an increased release of inflammatory cytokines and increased expression of NF-κB over time. Moreover, H_2_ administration produced concomitant declines in the release of inflammatory cytokines and in the expression of p-Akt and NF-κB. Therefore, the Akt/NF-κB signalling pathway may mainly affect the inflammatory regulation of H_2_ during burn wound progression. However, the described indexes and proteins associated with apoptosis and autophagy expressed opposite variation trends at across various time windows after burn or with HS management, compared with p-Akt expression. Hence, we cannot argue a link between Akt signals and the regulation of apoptosis and autophagy. During burn wound progression, other signalling pathways may be involved that directly control apoptosis and autophagy.

## Conclusion

In conclusion, the present study demonstrates the beneficial effect of H_2_ on burn wound progression by attenuating oxidative stress and inhibiting apoptosis and inflammation. The Akt/NF-κB signalling pathway is involved in the regulation of H_2_ to release inflammatory cytokines. These results may also indicate that HS is a potential therapy to prevent wound deepening and extension during the early stage of burns, considering its efficacy, safety, and convenience.

## Supporting Information

S1 Fig The result of total protein quantification indicated the equal protein levels of loaded samples from different groups. (TIF)
