# Supplementary figures and images for: Beneficial Effects of Hydrogen-Rich Saline on Early Burn-Wound Progression in Rats
Source: PLoS One. 2015 Apr 13;10(4):e0124897. doi: 10.1371/journal.pone.0124897 (PMC4395383; doi:10.1371/journal.pone.0124897)

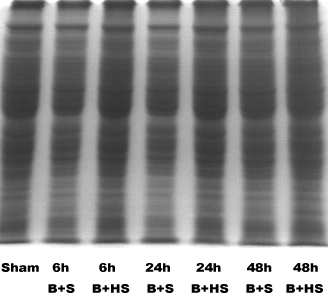

Supplement: S1 Fig — (TIF) [file pone.0124897.s001.tif]
